# Sexual Orientation Disparities in BMI among US Adolescents and Young Adults in Three Race/Ethnicity Groups

**DOI:** 10.1155/2014/537242

**Published:** 2014-04-29

**Authors:** Sabra L. Katz-Wise, Emily A. Blood, Carly E. Milliren, Jerel P. Calzo, Tracy K. Richmond, Holly C. Gooding, S. Bryn Austin

**Affiliations:** ^1^Division of Adolescent and Young Adult Medicine, Boston Children's Hospital, Boston, MA, USA; ^2^Department of Pediatrics, Harvard Medical School, Boston, MA, USA; ^2^Geisel School of Medicine, Dartmouth College, Hanover, NH, USA; ^4^Department of Social and Behavioral Sciences, Harvard School of Public Health, Boston, MA, USA

## Abstract

Obesity is a key public health issue for US youth. Previous research with primarily white samples of youth has indicated that sexual minority females have higher body mass index (BMI) and sexual minority males have lower BMI than their same-gender heterosexual counterparts, with sexual orientation differences in males increasing across adolescence. This research explored whether gender and sexual orientation differences in BMI exist in nonwhite racial/ethnic groups. Using data from Waves I–IV (1995–2009) of the US National Longitudinal Study of Adolescent Health (*N* = 13,306, ages 11–34 years), we examined associations between sexual orientation and BMI (kg/m^2^) over time, using longitudinal linear regression models, stratified by gender and race/ethnicity. Data were analyzed in 2013. Among males, heterosexual individuals showed greater one-year BMI gains than gay males across all race/ethnicity groups. Among females, white and Latina bisexual individuals had higher BMI than same-race/ethnicity heterosexual individuals regardless of age; there were no sexual orientation differences in black/African Americans. Sexual orientation disparities in BMI are a public health concern across race/ethnicity groups. Interventions addressing unhealthy weight gain in youth must be relevant for all sexual orientations and race/ethnicities.

## 1. Introduction

Obesity is a key public health issue for US youth, particularly among specific sociodemographic groups, including some racial/ethnic and sexual orientation groups [1, 2]. Obesity is operationalized as having a body mass index (BMI) equal to or greater than the 95th percentile among individuals younger than age 18 years or a BMI of 30 or greater for individuals age 18 years or older [3]. Previous research in a primarily white cohort of youth and young adults, age 12–23 years, found that sexual minority (nonheterosexually identified) females had higher BMI than heterosexual females throughout adolescence [4], similar to patterns seen in adult females [5]. Among males in this cohort, gay males had higher BMI in early adolescence compared to heterosexual males, but by late adolescence BMI among gay males was lower than their heterosexual peers [4], similar to patterns seen in adult males [6]. However, little is known about the intersection of race/ethnicity and sexual orientation and its impact on youth weight status.

A small number of studies have investigated sexual orientation patterns in BMI among multiethnic samples of adults [7, 8]. One such study found that among females, white and African American sexual minorities were at increased risk of being overweight compared to same-race/ethnicity heterosexual individuals, whereas among adult males, gay males were less likely than heterosexuals to be overweight among white, African American, Asian, and Latino men [7]. We are aware of only one study with a representative sample of adolescents examining sexual orientation disparities in BMI in a multiethnic sample, which found that bisexual female and male youth were at elevated risk for obesity compared to same-gender heterosexual youth across race/ethnicity groups [9]. However, no research has explored whether an age-by-orientation interaction effect exists in racial/ethnic minority youth.

Disparities in BMI among sexual minorities have been explained primarily using the minority stress model, which suggests that experiences of prejudice and discrimination based on minority status negatively affect health [10]. Sexual minorities who are also racial/ethnic minorities may be at greater risk for negative health outcomes due to experiences of minority stress based on being a member of multiple minority groups [11, 12]. Individuals may cope with minority stress by engaging in unhealthy weight-related behaviors. Indeed, research on sexual orientation, body image, and eating disorders in primarily white samples of adults has suggested that compared with heterosexuals, gay males indicated greater body dissatisfaction and eating disorder symptomatology [13, 14].

An alternative explanation is that sexual orientation disparities in BMI are related to sociocultural ideals regarding body appearance. For instance, sexual minority male youth reported greater desire for muscularity, but fewer attempts to gain weight, compared to heterosexual male youth [15]. Among adult females, lesbian and bisexual individuals indicated lower internalization of sociocultural appearance ideals for a thin body type compared to heterosexual females [14]. These findings may help to explain why sexual minority females have higher BMI and sexual minority males have lower BMI, compared to their same-gender heterosexual counterparts. However, similar to research on sexual orientation-by-gender disparities in obesity, this research was conducted with primarily white samples. More research is needed to first identify whether sexual orientation-by-gender disparities in obesity exist in nonwhite racial/ethnic groups and then to examine whether explanations for these disparities apply across racial/ethnic groups.

Previous obesity prevention and intervention efforts have been only marginally successful, in part because they tend not to be appropriately tailored and instead use a* one size fits all* approach. In a recent review of school-based Internet obesity prevention programs for adolescents, a number of programs targeted racial/ethnic minorities who are at greater risk for obesity and the majority of programs included content on nutrition and physical activity [16]. However, none of the programs reviewed seemed to address issues related to sexual orientation and obesity, such as body image or sociocultural ideals of thinness and muscularity. More research is needed to identify subgroups most at risk for obesity by determining whether sexual orientation-by-gender disparities exist across race/ethnicity groups, such that intervention and prevention efforts can be more effectively tailored for these groups.

The transition from adolescence to young adulthood is a critical period for weight gain and the development of obesity, with long-term negative health implications for excessive weight gain during young adulthood [17, 18]. In addition, previous research has indicated that associations between sexual orientation and BMI change across adolescence and into young adulthood [4]. Longitudinal research with nationally representative samples of adolescents is needed to address whether age-by-sexual orientation effects exist among nonwhite youth. To address this question and to inform obesity prevention and weight-loss intervention efforts, the current study used longitudinal data from Waves I–IV of the National Longitudinal Study of Adolescent Health (Add Health) to examine sexual orientation disparities in BMI over time within female and male race/ethnicity groups. Specific sexual minority subgroups were compared separately to heterosexual individuals because previous research has found BMI and obesity prevalence to differ among these subgroups, with bisexual individuals at particularly high risk for elevated BMI and obesity [9, 19]. We hypothesized that female sexual minorities, particularly bisexual individuals, would have consistently higher BMI over time than heterosexual females. We further hypothesized that heterosexual males would experience greater one-year increases in BMI compared to gay males. Finally, we hypothesized that these patterns would be similar across all three racial/ethnic groups.

## 2. Materials and Methods

### 2.1. Study Sample

After exclusion criteria were applied (described below), the current sample included 7,140 females and 6,166 males, who contributed data to at least one of the four waves of Add Health, a US nationally representative longitudinal cohort [20]. Participants were age 11–21 years at Wave I (1995) and age 24–34 years at Wave IV (2008-2009). Analyses were restricted to participants who provided a report of sexual orientation identity at Wave III and self-identified as non-Latino white (59%), non-Latino black/African American (23%), and Latino (18%) at Wave I. Other race/ethnicity groups were excluded due to a small sample size within some sexual orientation groups. Descriptive statistics for age and BMI by race/ethnicity, gender, and sexual orientation are reported in Table 1. This study was approved by the Boston Children's Hospital Institutional Review Board.

### 2.2. Measures

Sexual orientation identity was assessed at Wave III with one item asking participants to choose the description that best fits how they think about themselves, with the following response options:* 100% heterosexual (straight); mostly heterosexual (straight), but somewhat attracted to people of your own sex; bisexual*,* that is, attracted to men and women equally; mostly homosexual (gay), but somewhat attracted to people of the opposite sex; 100% homosexual (gay); not sexually attracted to either males or females.* Gender was assessed at Wave I as* female *or* male*. Race and ethnicity were assessed separately at Wave I but recoded and combined into the following groups for analysis:* non-Latino white, non-Latino black/African American, and Latina/o*. Age in years and age-specific BMI (kg/m^2^) calculated from self-reported height and weight were assessed at each wave. Self-reported height and weight were used because measured height and weight were not available at all four waves.

### 2.3. Statistical Analysis

To test the hypotheses, we conducted longitudinal unweighted linear generalized estimating equation analyses in SAS (version 9.3; Cary, NC). Data were analyzed in 2013. Analyses were stratified by gender and race/ethnicity, with* heterosexual *as the reference group. For the current study, participants who responded that they were not sexually attracted to either gender were excluded from the analyses, and mostly homosexual and 100% homosexual were combined into* lesbian/gay *due to small sample sizes, yielding the following sexual orientation identity groups:* heterosexual, mostly heterosexual, bisexual, and lesbian/gay. *To address the nonlinearity of BMI across development [21–23], age was modeled both linearly and quadratically and sexual orientation-by-age was used to model repeated measures of continuous BMI across ages 11–34 years, with age and BMI updated at each wave.

Weights are typically used in analysis of data from Add Health to allow for population estimates. We conducted unweighted analyses because the complexity of the models in examining BMI trajectories across waves and accounting for clustering by schools did not allow for the incorporation of weights. In addition, a model-based analysis is reasonable if design effects are taken into account [24], which the current analysis did by adjusting for gender, race/ethnicity, and age.

## 3. Results

Sexual orientation and race/ethnicity group differences in mean age at each wave were found. Among females, bisexuals and mostly heterosexual individuals were significantly younger (bisexual range: 0.33 to 0.43 years; mostly heterosexual range: 0.25 to 0.30 years) than completely heterosexual individuals at all waves, *P* < 0.02 to *P* < 0.0001. No significant sexual orientation group differences were found among males for mean age at each wave. Among both females and males, Latinos were significantly older (female range: 0.43 to 0.49 years; male range: 0.36 to 0.44 years) than same-gender non-Latinos at all waves, *P* < 0.0001. In addition, non-Latina black/African American females were significantly older (0.15 years) than non-Latina white females at Wave II only, *P* < 0.01.

**Figure 1:**
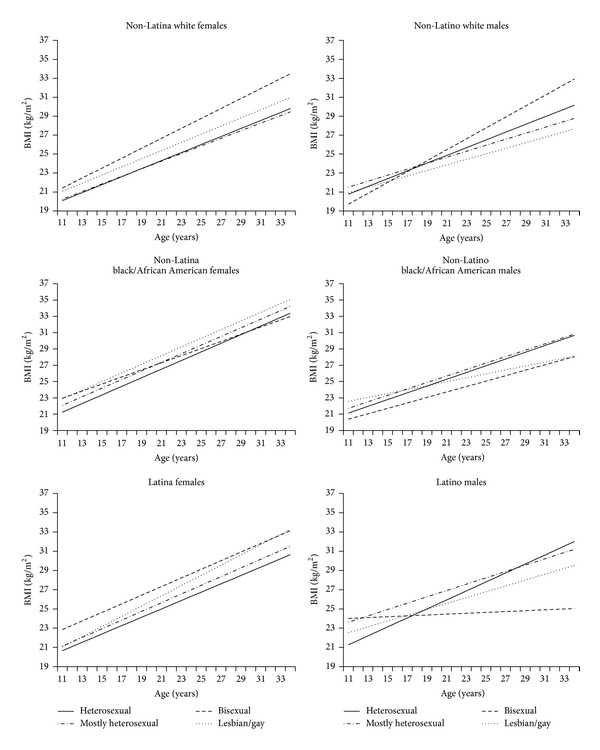
BMI trajectories by sexual orientation across race/ethnicity groups of adolescents and young adults in the US National Longitudinal Study of Adolescent Health, Waves I–IV (1995–2009).

Descriptively, among both females and males across sexual orientation and race/ethnicity groups, age-specific BMI increased substantially across time from age 11 to 34 years (Table 1, Figure 1). Among females, the association between sexual orientation and BMI did not differ significantly by age, so sexual orientation-by-age interaction terms were not included in the final models. Non-Latina white and Latina bisexual individuals had higher BMI compared to their heterosexual female counterparts, while no sexual orientation differences were observed among non-Latina black/African American females (see Table 2, Figure 1).

Among males, the association between sexual orientation and BMI differed significantly by age within each of the three race/ethnicity groups. Gay males had higher BMI than heterosexual males in early adolescence. However, heterosexual males showed greater one-year BMI gains over time surpassing gay males by approximately age 17 years, with disparities widening further as participants aged into adulthood (see Table 2, Figure 1). Bisexual individuals showed a different pattern, with bisexual males showing greater one-year BMI gains over time compared to heterosexual males, but only among non-Latino white participants.

## 4. Discussion

Previous research with a predominantly white cohort of youth found that age modified sexual orientation disparities in BMI in males. The current research extended these findings to non-Latino black/African American and Latino young men. During adolescence and young adulthood, heterosexual males demonstrated greater yearly increases in BMI compared to gay males, putting them at excess risk for obesity. It is not clear why these patterns are emerging, but reporting bias could be one factor. A prior Add Health analysis found that gay males underreport their BMI by an estimated 0.37 BMI units more than heterosexual males [25]; nevertheless, bias of this magnitude would not be sufficiently large to explain the differences observed in the current study. Another potential explanation for smaller increases in BMI among gay males may be that compared to heterosexual males, gay males are at greater risk for body dissatisfaction and eating disorder symptomatology, which may result in lower BMI over time [13, 14]. Other research has suggested that sexual minority male adolescents and young adults are less likely to attempt to gain weight compared to completely heterosexual male youth [15], which may represent a protective factor against the development of obesity among sexual minority male youth.

This study also found higher BMI among bisexual non-Latina white and Latina females compared to same-race/ethnicity heterosexual females, but not in other sexual minority female subgroups. It is possible that bisexual females may be responding to sexual minority stressors (e.g., increased rates of victimization) [26] by engaging in obesogenic behaviors (e.g., stress-induced binge eating) [27], more so than other sexual minority females or gay males. Higher BMI among bisexual females may also be attributable to comorbidity of obesogenic behaviors with other health risk behaviors and negative health outcomes. For instance, other research has indicated that bisexual females are at greater risk for psychological distress [28] and health risk behaviors, including substance use [29] and self-injurious behavior [30], compared to other sexual orientation groups. A recent study found that compared to lesbians, bisexual women are more likely to use maladaptive coping strategies, which may explain more adverse mental and physical health outcomes in bisexual females compared to lesbian females [28]. Results from the current study highlight the need for research on health outcomes within sexual minority subgroups, in addition to comparing sexual minorities with completely heterosexual individuals. In addition, more research is needed to understand why bisexual females and males and heterosexual males have greater risk for increased BMI and whether membership in other sexual orientation groups may confer specific protective factors against weight gain and development of obesity.

## 5. Conclusions

Findings from this study demonstrated that sexual orientation and gender differences in BMI are not limited to non-Latino white youth and young adults. Among males, heterosexual males showed greater one-year BMI gains than gay males across all race/ethnicity groups. Among females, non-Latina white and Latina bisexual individuals had higher BMI than same-race/ethnicity heterosexual individuals regardless of age; there were no sexual orientation differences in non-Latina black/African Americans. It is clear from these results that sexual orientation disparities in BMI are a public health concern across race/ethnicity groups. Obesity prevention and intervention efforts should target healthy body image and weight-management methods for all youth, but additional resources may be needed for sexual minority youth. In particular, interventions should be designed in such a way as to not exacerbate risk of unhealthy weight control behaviors and eating disorders. In summary, obesity prevention initiatives and treatment interventions addressing unhealthy weight gain in adolescence and young adulthood must be relevant for all sexual orientations and race/ethnicities.

## Figures and Tables

**Table 1:** Descriptive statistics^a^ for age^b^ and BMI^c^ by race/ethnicity, gender, and sexual orientation among 7,140 female and 6,166 male adolescents and young adults in the US National Longitudinal Study of Adolescent Health, Waves I–IV (1995–2009).

	Non-Latino white							
	Females				Males			
	Heterosexual (*n* = 12,500)	Mostly heterosexual (*n* = 2,815)	Bisexual (*n* = 354)	Lesbian (*n* = 256)	Heterosexual (*n* = 13,143)	Mostly heterosexual (*n* = 574)	Bisexual (*n* = 98)	Gay (*n* = 325)
Age (Wave I)	15.97 (1.72)	15.74 (1.70)	15.57 (1.55)	15.87 (22.73)	16.15 (1.70)	15.91 (1.73)	16.05 (1.48)	15.88 (1.64)
BMI								
Wave I	21.84 (4.10)	21.79 (4.11)	22.97 (5.31)	22.73 (5.07)	22.59 (4.37)	22.55 (5.25)	22.38 (3.86)	21.90 (3.93)
Wave II	22.20 (4.48)	22.14 (4.35)	24.18 (6.36)	23.56 (5.33)	22.96 (4.41)	23.11 (5.85)	23.30 (4.08)	22.94 (4.41)
Wave III	24.98 (5.96)	24.91 (5.69)	27.98 (7.77)	26.16 (6.67)	25.78 (5.10)	24.57 (5.85)	25.86 (5.17)	24.19 (4.59)
Wave IV	27.47 (7.17)	27.07 (6.87)	30.25 (9.40)	28.52 (7.29)	27.96 (5.77)	27.04 (7.43)	30.17 (6.34)	29.06 (5.28)

	Non-Latino black/African American							
	Females				Males			
	Heterosexual (*n* = 5,481)	Mostly heterosexual (*n* = 704)	Bisexual (*n* = 154)	Lesbian (*n* = 153)	Heterosexual (*n* = 4,690)	Mostly heterosexual (*n* = 106)	Bisexual (*n* = 30)	Gay (*n* = 125)

Age (Wave I)	16.02 (1.70)	15.84 (1.70)	15.34 (1.75)	15.62 (1.88)	16.08 (1.74)	16.15 (1.80)	15.29 (1.79)	15.60 (1.62)
BMI								
Wave I	23.53 (5.11)	24.08 (4.95)	24.85 (6.02)	24.86 (5.44)	22.93 (4.39)	23.47 (5.37)	21.93 (3.92)	23.07 (5.29)
Wave II	23.95 (5.47)	24.72 (5.16)	25.67 (7.21)	25.56 (6.27)	23.46 (4.63)	25.16 (5.75)	21.39 (2.67)	24.76 (6.40)
Wave III	27.17 (7.11)	28.29 (7.25)	27.90 (8.02)	28.79 (6.79)	26.27 (5.53)	26.42 (5.32)	25.71 (5.27)	25.38 (6.02)
Wave IV	30.54 (8.16)	31.16 (8.07)	30.47 (7.28)	32.14 (8.81)	28.45 (6.28)	28.75 (8.49)	26.20 (7.26)	26.72 (6.49)

	Latina/o							
	Females				Males			
	Heterosexual (*n* = 3,654)	Mostly heterosexual (*n* = 566)	Bisexual (*n* = 101)	Lesbian (*n* = 97)	Heterosexual (*n* = 3,719)	Mostly heterosexual (*n* = 121)	Bisexual (*n* = 38)	Gay (*n* = 155)

Age (Wave I)	16.44 (1.63)	16.00 (1.58)	16.07 (2.09)	15.83 (1.82)	16.47 (1.69)	16.22 (1.75)	16.25 (2.21)	16.52 (1.59)
BMI								
Wave I	22.79 (4.31)	22.96 (4.23)	24.47 (3.40)	23.39 (5.11)	23.55 (4.78)	24.90 (6.45)	23.43 (6.46)	24.15 (4.45)
Wave II	22.89 (4.37)	23.45 (5.46)	25.29 (4.04)	23.44 (4.50)	23.88 (4.76)	26.00 (6.69)	25.84 (7.72)	24.72 (5.27)
Wave III	25.93 (5.60)	26.62 (6.71)	28.68 (6.11)	28.18 (5.47)	27.15 (5.23)	29.19 (8.67)	25.81 (5.33)	25.20 (3.87)
Wave IV	28.46 (6.93)	29.01 (7.58)	30.72 (7.36)	29.65 (8.02)	29.65 (5.99)	29.21 (7.36)	24.49 (11.43)	28.37 (5.63)

^a^Means are reported with standard deviations in parentheses.

^b^Age is in years.

^c^BMI was measured as kg/m^2^.

**Table 2:** Sexual orientation differences in BMI^a^ in 7,140 female and 6,166 male adolescents and young adults in the US National Longitudinal Study of Adolescent Health, Waves I–IV (1995–2009).

Measure	Non-Latino white		Non-Latino black/African American		Latina/o	
	Female	Male	Female	Male	Female	Male
	*β* (95% CI)	*β* (95% CI)	*β* (95% CI)	*β* (95% CI)	*β* (95% CI)	*β* (95% CI)
Sexual orientation						
Heterosexual	(ref)	(ref)	(ref)	(ref)	(ref)	(ref)
Mostly heterosexual	−0.05 (−0.43, 0.32), 2815	−0.13 (−10.06, 0.81), 574	0.86 (−0.003, 1.72), 704	0.37 (−1.64, 2.37), 106	0.65 (−0.27, 1.57), 566	1.09 (−1.04, 3.21), 121
Bisexual	**2.32 (0.97, 3.68)** ^ b^, 354	0.52 (−1.13, 2.17), 98	0.92 (−0.95, 2.78), 154	−1.48 (−4.08, 1.12), 30	**2.37 (0.58, 4.17)**, 101	−1.17 (−4.89, 2.55), 38
Lesbian/gay	1.06 (−0.23, 2.35), 256	**−0.94 (−1.82, −0.07)**, 325	1.68 (−0.15, 3.51), 153	−0.22 (−2.06, 1.63), 125	1.41 (−0.62, 3.44), 97	−0.31 (−1.66, 1.04), 155
Age^c^	**0.45 (0.44, 0.47)**	**0.46 (0.45, 0.48)**	**0.57 (0.55, 0.60)**	**0.48 (0.45, 0.50)**	**0.49 (0.46, 0.52)**	**0.53 (0.51, 0.56)**
Age^2c^	**−0.01 (−0.01, −0.01)**	**−0.01 (−0.02, −0.01)**	**−0.01 (−0.02, −0.01)**	**−0.02 (−0.02, −0.01)**	**−0.01 (−0.02, −0.01)**	**−0.01 (−0.02, −0.01)**
Sexual orientation × age						
Heterosexual	—	(ref)	—	(ref)	—	(ref)
Mostly heterosexual	—	**−0.10 (−0.15, −0.05)**	—	−0.01 (−0.20, 0.17)	—	−0.14 (−0.29, 0.001)
Bisexual	—	**0.17 (0.02, 0.31)**	—	−0.10 (−0.35, 0.16)	—	**−0.43 (−0.72, −0.15)**
Gay	—	**−0.12 (−0.18, −0.07)**	—	**−0.19 (−0.28, −0.11)**	—	**−0.16 (−0.26, −0.06)**

^a^BMI was measured as kg/m^2^.

^b^Significant effects are bolded.

^c^Age and age^2 ^were centered on the mean age in the sample: 20.5 years.
